# Post-Translational Control of IL-1β via the Human Papillomavirus Type 16 E6 Oncoprotein: A Novel Mechanism of Innate Immune Escape Mediated by the E3-Ubiquitin Ligase E6-AP and p53

**DOI:** 10.1371/journal.ppat.1003536

**Published:** 2013-08-01

**Authors:** Martina Niebler, Xu Qian, Daniela Höfler, Vlada Kogosov, Jittranan Kaewprag, Andreas M. Kaufmann, Regina Ly, Gerd Böhmer, Rainer Zawatzky, Frank Rösl, Bladimiro Rincon-Orozco

**Affiliations:** 1 Division of Viral Transformation Mechanisms, German Cancer Research Center (DKFZ), Heidelberg, Germany; 2 Gynecological Tumor-Immunology, Charité Campus Benjamin Franklin, Berlin, Germany; 3 Division of Genome Modifications and Carcinogenesis, German Cancer Research Center (DKFZ), Heidelberg, Germany; 4 Molecular Medicine Program, Faculty of Science, Mahidol University, Bangkok, Thailand; 5 Deutsche Klinik Bad Münder, Hannover, Germany; Harvard Medical School, United States of America

## Abstract

Infections with high-risk human papillomaviruses (HPVs) are causally involved in the development of anogenital cancer. HPVs apparently evade the innate immune response of their host cells by dysregulating immunomodulatory factors such as cytokines and chemokines, thereby creating a microenvironment that favors malignancy. One central key player in the immune surveillance interactome is interleukin-1 beta (IL-1β) which not only mediates inflammation, but also links innate and adaptive immunity. Because of its pleiotropic physiological effects, IL-1β production is tightly controlled on transcriptional, post-translational and secretory levels. Here, we describe a novel mechanism how the high-risk HPV16 E6 oncoprotein abrogates IL-1β processing and secretion in a NALP3 inflammasome-independent manner. We analyzed IL-1β regulation in immortalized keratinocytes that harbor the HPV16 E6 and/or E7 oncogenes as well as HPV-positive cervical tumor cells. While in primary and in E7-immortalized human keratinocytes the secretion of IL-1β was highly inducible upon inflammasome activation, E6-positive cells did not respond. Western blot analyses revealed a strong reduction of basal intracellular levels of pro-IL-1β that was independent of dysregulation of the NALP3 inflammasome, autophagy or lysosomal activity. Instead, we demonstrate that pro-IL-1β is degraded in a proteasome-dependent manner in E6-positive cells which is mediated via the ubiquitin ligase E6-AP and p53. Conversely, in E6- and E6/E7-immortalized cells pro-IL-1β levels were restored by siRNA knock-down of E6-AP and simultaneous recovery of functional p53. In the context of HPV-induced carcinogenesis, these data suggest a novel post-translational mechanism of pro-IL-1β regulation which ultimately inhibits the secretion of IL-1β in virus-infected keratinocytes. The clinical relevance of our results was further confirmed in HPV-positive tissue samples, where a gradual decrease of IL-1β towards cervical cancer could be discerned. Hence, attenuation of IL-1β by the HPV16 E6 oncoprotein in immortalized cells is apparently a crucial step in viral immune evasion and initiation of malignancy.

## Author Summary

Persistently high-risk HPV-infected individuals have an increased risk to develop anogenital cancer. HPV encodes the viral proteins E6 and E7 that interact with and induce the degradation of the cell cycle regulators p53 and pRb, respectively, priming immortalized keratinocytes towards malignant transformation. In early antiviral immune response, IL-1β is an important factor for the initiation of inflammation and activation of immune cells such as macrophages and T cells. Our study describes a post-translationally controlled pathway where E6 mediates proteasomal degradation of IL-1β in HPV16-immortalized human keratinocytes. This process depends on the cellular ubiquitin ligase E6-AP and p53 highlighting a novel molecular mechanism of a virus-host interaction that is critical for evading innate immune defense. IL-1β dysregulation is also found in tissue sections which represent different stages of virus-induced carcinogenesis, underlining the clinical relevance of our findings.

## Introduction

High-risk human papillomaviruses (HPVs) are causally responsible for anogenital cancer, both in women and men [1], [2]. While in the latter, penile and anal carcinomas are relatively rare, HPV infection is also linked in both genders to more than 50% of all oropharyngeal squamous cell carcinomas [1], [3], [4]. The transforming potential of these viruses is mediated by the E6 and E7 oncoproteins that are responsible for sustaining a proliferative phenotype mainly by promoting degradation of the cellular tumor suppressor proteins p53 and pRb, respectively [1], [2].

During the last years, however, it became evident that viral oncoproteins not only affect cell cycle regulatory mechanisms and apoptosis, but also have a negative impact on the innate immune response of their host and in turn on the respective premalignant microenvironment where unscheduled growth of persistently infected cells is finally taking place [5], [6]. Monitoring chemotactic and pro-inflammatory genes in a top-down approach, genome-wide transcriptome analyses and the subsequent *in silico* topological reconstruction of the cellular immune network shows that high-risk HPVs always target highly interconnected nodes of the antiviral defense interactome, leading either to cell lysis and virus spread, to viral persistence or ultimately to malignant transformation [5]–[9]. In other words, considering virus-host interactions as a result of a long-lasting evolutionary selection process, HPVs have developed sophisticated strategies to circumvent innate immunity long before the adaptive immune response is activated [10]–[12].

Concerning our understanding about the role of the individual oncoproteins and their cross-talk with the host cell interactome, E6 and E7 either directly or indirectly interfere with innate immunosurveillance [5], [6], [11]. For instance, the high-risk HPV E6 oncoprotein both inactivates type I interferon (IFN) signaling (e.g. keratinocyte-specific IFN-κ) [13] and downstream pathways such as chemokine expression which is required to attract and activate specific subsets of effector leukocytes, cells from the monocyte/macrophage lineage as well as natural killer cells [14]. E7 expression, on the other hand, can inhibit the function and nuclear translocation of p48 (ISGF3γ), one component of the IFN-stimulated gene factor 3 (ISGF3) trimeric complex formed between p48, STAT1 and STAT2. If p48 is missing, the transcription of IFN-regulated genes via its binding to cognate IFN-stimulated response elements is also abrogated [15].

Another key player that is located in the center of the antiviral and pro-inflammatory network is interleukin-1β (IL-1β) [16]. This cytokine is a potent activator of immune responses directed against viral and bacterial infections [17]. It mediates the migration of leukocytes, induces fever and promotes the activation and polarization of T cells [18]. While the precursor protein of IL-1α (pro-IL-1α) is cleaved by calpain, pro-IL-1β is processed by caspase-1 to its biologically active secreted form [18], [19]. The activating caspase-1 in turn is a component of a multi-protein complex, called NALP3 [NACHT-, leucine-rich repeat (LRR)-and PYD-containing protein 3] inflammasome that consists of the NOD-like receptor protein NALP3, the adaptor protein ASC (apoptosis-associated speck-like protein containing a caspase recruitment domain) and pro-caspase-1 [20]. NALP3 inflammasome assembly and the subsequent autoproteolytic cleavage of pro-caspase-1 is not only activated upon viral or bacterial infection [20]–[22], but also by numerous exogenous and endogenous danger signals such as extracellular ATP (a known inducer of potassium efflux via the P2X7 receptor [23]), hyaluronan [24], amyloid-β fibrils [25] and intracellular reactive oxygen species (ROS) [26]. While IL-1α is mainly found to be membrane-associated, IL-1β is active in an auto-/paracrine manner when secreted [16], [18]. Mature IL-1β and IL-1α share the same IL-1 receptor which is constitutively expressed on many cell types. Upon binding, NF-κB and the mitogen-activated protein (MAP) kinases p38 and JNK are activated to induce a wide range of genes involved in the pro-inflammatory response [16], [18], [27]. Abnormal IL-1β release or lack of IL-1β expression changes the conditions towards a pathological microenvironment, resulting either in chronic inflammation or in the absence of a proper immune surveillance against infections, respectively [16], [18], [19], [28].

Apart from immunological effector cells such as neutrophils, macrophages and dendritic cells, also keratinocytes are a potent source of IL-1β [29], [30]. These cells are important sensors of pathogens and danger signals that mediate immune responses, supporting the notion that keratinocytes, which are the main target of HPV infection, also have to be considered as central nonprofessional immune competent cells of the mucosa [10], [31]. Although IL-1β was recently identified to be targeted by high-risk HPV as a central hub within the network of innate immunity [5] and down-regulated in cervical tumors [32]–[34], our knowledge about its function and regulation in the context of HPV-induced carcinogenesis is still rudimentary.

To get insights on how this central cytokine is regulated, we monitored the processes that modulate the NALP3 inflammasome complex and in turn IL-1β secretion using primary and HPV16-immortalized human keratinocytes as well as HPV-positive cervical carcinoma cells as experimental model system. Although the *IL1B* gene is still transcribed in non-malignant cells, the protein levels of pro-IL-1β as well as the secretion of mature IL-1β is strongly impaired in E6- and E6/E7- immortalized keratinocytes. Excluding different pathways regulating protein degradation (e.g. via lysosomes or autophagy), finally we show that the E3 ubiquitin ligase E6-AP ( = E6-associated protein) and p53 are controlling proteasomal degradation of pro-IL-1β in high-risk HPV-positive cells. We conclude that an inflammasome-independent, but proteasome-mediated degradation of pro-IL-1β represents an early viral immune evasion mechanism occurring during high-risk HPV immortalization, ultimately culminating in the complete silencing of the gene itself in malignant cells. Additionally, transcriptional silencing of the *IL1B* gene also seems to be a favored mode of escape in hyperproliferative cells infected by HPV6 and HPV11, where post-translational degradation of IL-1β was much less efficient. Since a gradual absence of IL-1β was also found in cervical tissue sections, inactivation of IL-1β signaling apparently inhibits its central role in the balance between inflammation and antiviral immunity against an HPV infection, thereby significantly contributing to the development of cervical cancer.

## Results

### HPV16 E6-positive cells show impaired IL-1β secretion after viral infection

**Figure 1:**
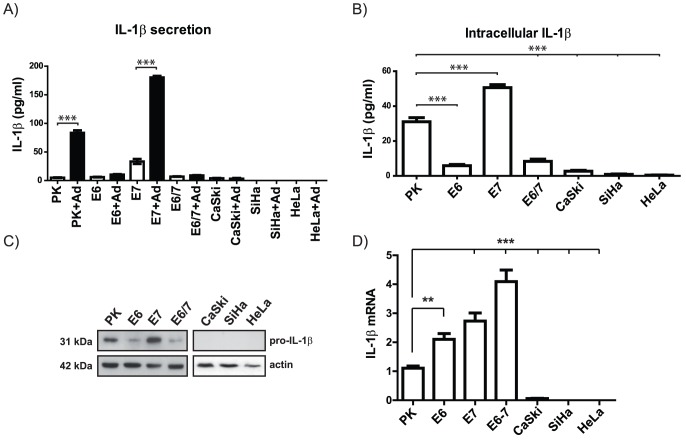
Expression and secretion of IL-1β in human primary keratinocytes and HPV-positive cell lines. A) Quantification of secreted IL-1β (expressed as pg/ml) by ELISA in human primary keratinocytes (PK), keratinocytes immortalized by individual oncogenes (E6, E7, E6/E7) and HPV16/18-positive cervical carcinoma cell lines (SiHa, CaSki, HeLa, respectively) 24 h after infection with a recombinant GFP-expressing adenovirus 5 (Ad) in comparison to uninfected cells. B) Quantification of basal intracellular IL-1β levels by ELISA. C) Western blot analysis of pro-IL-1β in human primary keratinocytes (PK) and HPV-positive cells. Actin was used as a loading control. D) qPCR analysis of IL-1β cDNA obtained from PK and HPV-positive cells (Ordinate: expressed as fold changes using the average IL-1β steady state level of 3 different primary human keratinocyte preparations PK cells as a reference which was arbitrarily set as 1. The graphs in A, B and D represent the mean values (± SEM) of three independent experiments. ANOVA ***p<0.05.

In order to analyze the influence of high-risk HPV on the capacity to express and secrete mature IL-1β in the context of different transformation stages of an HPV infection, we used primary human keratinocytes (PK), immortalized PK encoding individual oncoproteins of HPV16 (immE6, immE7, and immE6/E7) as well as HPV16/18-positive cervical tumor cells (CaSki, SiHa and HeLa). The cells were incubated with recombinant GFP-expressing adenoviruses, which are known to induce strong IL-1β secretion via activation of the NALP3 inflammasome [21], [35]–[37]. As shown in Fig. 1A, both PK and immE7 responded effectively by releasing mature IL-1β after a one hour exposure to adenoviral infection. ImmE6 and immE6/E7 cells only released small amounts of IL-1β, while cervical carcinoma cell lines completely failed to secrete IL-1β. The deficiency in mature IL-1β release in these cells was not due to reduced or missing adenovirus infectivity, since similar fluorescence levels of GFP could be detected in all cell lines 24 h post infection (Fig. S1A). Additionally, to test the biological activity of the secreted IL-1β in functional terms, human umbilical vein endothelial cells (HUVEC) were incubated for 12 hours with conditioned medium obtained from adenovirus-infected and non-infected cells. Here, only supernatants from infected immE7 keratinocytes contained biologically active IL-1β as demonstrated by substantial induction of IL-1β-responsive genes such as IL-6 and CCL-20 in HUVEC cells used as indicator cell line (Fig. S1B) [38], [39].

To evaluate whether the lack of IL-1β secretion was the result of a diminished synthesis of the protein itself, the intracellular levels of IL-1β were monitored by ELISA. Here, a strong reduction of IL-1β in immE6 and immE6/E7 cells and its complete absence in cervical carcinoma cell lines could be discerned (Fig. 1B). Since the ELISA did not discriminate between pro-IL-1β and its mature form, Western blot analysis was performed. As depicted in Fig. 1C, only PK and immE7 cells constitutively expressed substantial amounts of the 31 kDa pro-IL-1β form, while it was reduced in E6-positive immortalized keratinocytes or even absent in the tumor cell lines. When compared to primary human keratinocytes, parallel performed transcriptional analysis in immortalized cells by quantitative RT-PCR (q-PCR) even revealed an approximately two to four fold up-regulation of the basal pro-IL-1β mRNA levels (see Discussion), while in the respective malignant cells CaSki, SiHa and HeLa (Fig. 1D) as well as in the HPV-negative cervical cancer cell line C-33 A (Fig. S1C), transcription was either strongly reduced or absent. Hence, when tumor progression occurs, cervical cancers apparently use an additional strategy to inhibit the inflammatory response by diminishing IL-1β expression at the transcriptional level via gene silencing. Moreover, while still transcribed in immortalized keratinocytes, the same transcriptional down-regulation was also detected in cervical carcinoma cells when other cytokines such as IL-1α, IL-18 and IL-33 were monitored by q-PCR (Fig. S1D).

### Dysfunction of caspase-1 does not account for impaired basal IL-1β expression in E6-positive cells

**Figure 2:**
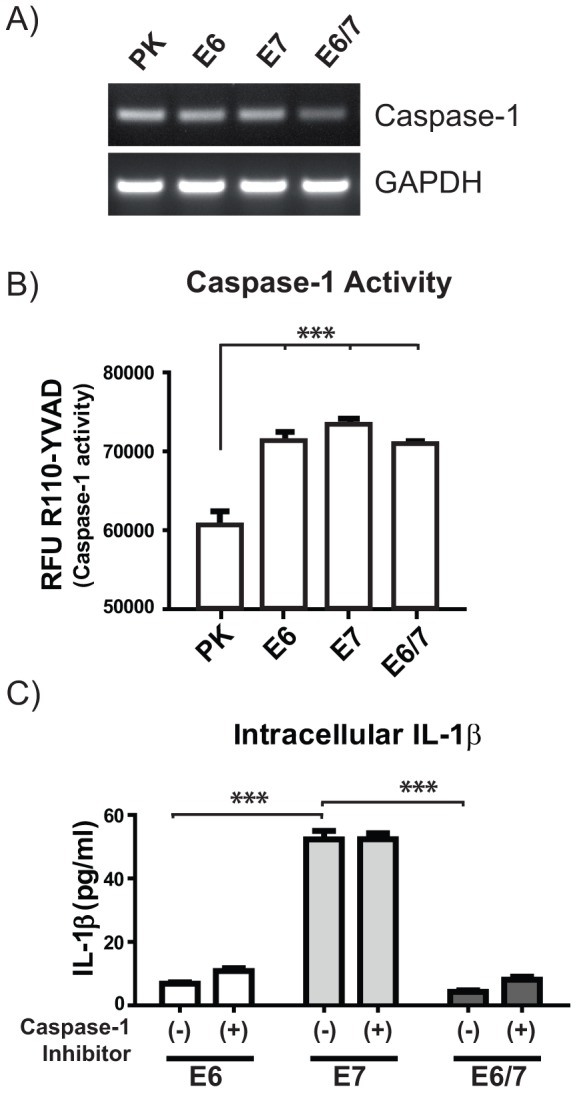
Caspase-1 activity did not account for decreased IL-1β in E6-positive cells. A) RT-PCR analysis of caspase-1 mRNA in comparison to the GAPDH steady state levels in PK and HPV-positive immortalized keratinocytes. B) Fluorometric measurement of caspase-1 activity was performed incubating the cells for 4 h at 37°C with 20 µM of the specific caspase-1 substrate R110-YVAD. RFU: Relative fluorescence units. C) Quantification of intracellular IL-1β levels by ELISA after 5 h of caspase-1 inhibition using 250 nM of caspase-1 inhibitor. The graphs in B and C represent the mean values (± SEM) of three independent experiments. ANOVA **p<0.01, ***p<0.001.

NALP3 inflammasome assembly, caspase-1 activation and subsequent IL-1β release can be triggered by a plethora of environmental insults, including viral infections [20]–[22]. Hence, a possibility that may explain the divergence in IL-1β levels and its release (Fig. 1A and B) could be a dysfunction in caspase-1 activity. Indeed, there are several reports that show that certain viruses, such as orthopoxviruses and influenza virus can interfere with signaling downstream of the inflammasome by modulating caspase-1 function [27]. Consistent with this notion is that caspase-1 knock-out mice have a higher susceptibility to various infections [17]. We therefore first examined the transcriptional levels of caspase-1 in PK and immortalized keratinocytes (Fig. 2A), where no substantial variances could be detected. Monitoring the basal activity of caspase-1 in PK and immortalized cells using the specific caspase-1 substrate R110-YVAD [40], again no significant alterations could be observed, although the activity was slightly higher in immortalized cells when compared to PK (Fig. 2B). On the other hand, since there exist naturally occurring genetic variants of human caspase-1 that can differ in their ability to activate IL-1β [41], we monitored whether inhibition of caspase-1 led to an increase of the intracellular levels of pro-IL-1β in immE6 and immE6/E7 cells. However, as depicted in Fig. 2C, this was not the case. Hence, these data suggest that the reduced levels of pro-IL-1β observed in immE6 and immE6/E7 cells are not the result of caspase-1 dysregulation.

### Enhanced basal levels of autophagy and lysosomal activity are not responsible for low pro-IL-1β levels in HPV16 E6- and E6/E7-immortalized keratinocytes

Autophagy is considered a master regulator of cellular homeostasis which is critical for survival under nutrient deprivation [42], [43]. Moreover, autophagy induction is able to diminish inflammation by targeting inflammasome components and pro-IL-1β for autophagosome/lysosomal degradation [20], [44]–[47]. Conversely, inhibition of autophagy potentiates inflammasome activity and prevents autophagosome/lysosome-mediated pro-IL-1β degradation [44]–[47]. A specific marker for autophagosome formation is the cytoplasmic microtubule-associated protein 1 light chain 3 (LC3-I) which is coupled to phosphatidylethanolamine to generate LC3-II [42], [43]. The latter accumulates at the inner membrane of the autophagosome to form so-called LC3-punctae which can be quantified by high throughput fluorescence microscopy analysis [48], [49].

**Figure 3:**
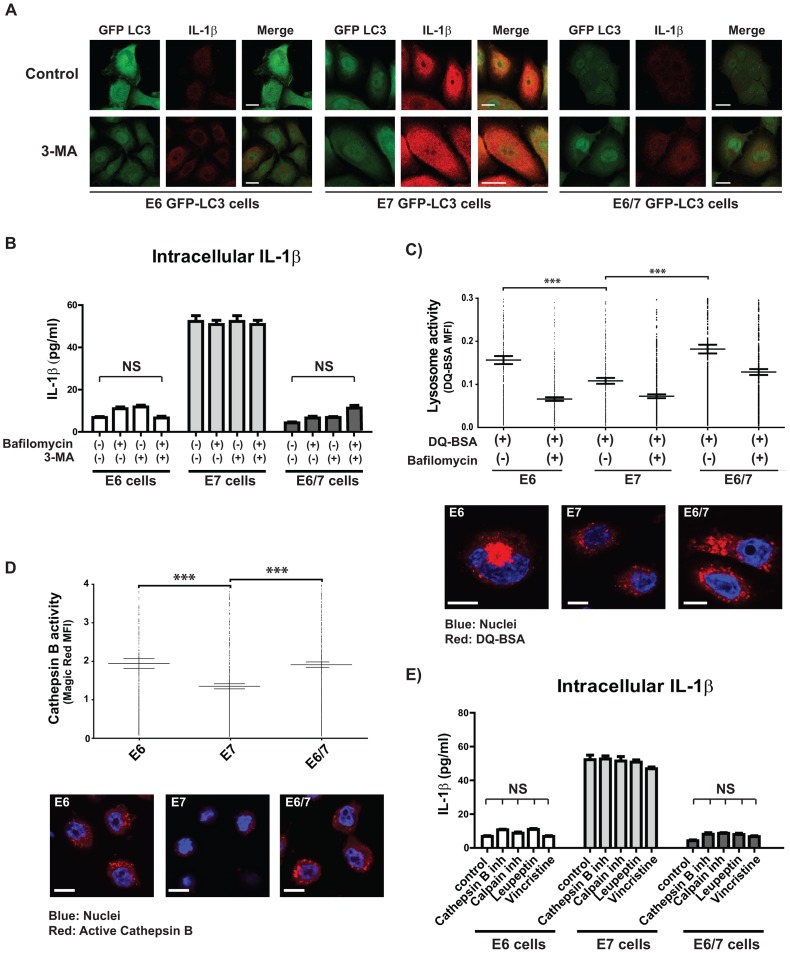
Decreased IL-1β levels observed in HPV16 E6-positive cells are independent of autophagy or lysosomal degradation. A) Confocal microscopy analysis after immunostaining of IL-1β (red) in immortalized keratinocytes after transduction with a GFP-tagged LC3-expressing lentivirus (LC3-GFP, green). GFP-LC3-positive cells were treated for 6 h with 1 mM 3-Methyladenine (3-MA) to block autophagy. The scale bars represent 10 µm. B) ELISA of intracellular IL-1β derived from immortalized HPV-positive cells treated for 6 h with 100 nM of bafilomycin to inhibit autophagosome maturation or with 1 mM of 3-MA to block autophagy. C) Quantification of the lysosomal activity (measured in mean fluorescence intensity, MFI) by confocal microscopy in immortalized HPV-positive cells. Cells were incubated with DQ™ Red-BSA for 8 h in the presence or absence of 100 nM bafilomycin. Nuclei (blue) were stained using Hoechst dye solution. The scale bar represents 10 µm. D) Quantification of cathepsin B activity (expressed as mean fluorescence intensity, MFI) by confocal microscopy in untreated HPV-positive cells stained with the specific fluorogenic cathepsin B substrate Magic Red™. Nuclei (blue) were stained using Hoechst dye solution. Scale bars represent 10 µm. E) ELISA of intracellular IL-1β from immortalized HPV-positive cells treated for 6 h with 25 µM cathepsin B inhibitor (CA-074 Me), 10 µM of lysosomal protease inhibitor (leupeptin), 20 µM of calpain inhibitor (PD150.606) or 20 µM of lysosome fusion inhibitor (vincristine). The analyses in C and D were performed using high throughput high resolution fluorescent microscopy analysis (BD pathway) in combination with a cell imaging analysis program (CellProfiler). The graphs show mean levels of five independent experiments each performed with 10.000 events/well per experiment (± SEM) ANOVA ***p<0.001. The bar graphs shown in B and E represent the standard error of the mean (± SEM) ANOVA ***p<0.001, NS: non-significant statistical differences.

We assessed basal autophagy by using a GFP-LC3 construct that was transduced into immortalized keratinocytes by lentiviral gene transfer. The number of LC3-punctae was then quantified in the presence of bafilomycin which stabilizes LC3-II within the autophagosomes by preventing their acidification [48], [49]. Here, significantly increased levels of LC3-punctae in immE6 and immE6/E7 cells were noticed (Fig. S2A). However, treatment with bafilomycin or 3-Methyladenin (3-MA) which is known to prevent autophagy via the inhibition of type III phosphatidylinositol 3-kinases (PI-3K) [50], did not recover intracellular levels of IL-1β in E6-positive cells as depicted by confocal microscopy (Fig. 3A) or ELISA (Fig. 3B). This observation revealed that the reduced protein levels of pro-IL-1β in immE6 and immE6/E7 are not a consequence of higher basal autophagocytic activity. Consistent with previous reports [44], [45], also experimental induction of autophagy by starvation demonstrated that pro-IL-1β is degraded in all cell lines (Fig. S2B).

Additionally, we addressed whether enhanced basal lysosomal activity may contribute to increased pro-IL-1β degradation in immE6 and immE6/E7 cells, since the maturation of autophagosomes is a step-wise process that finally culminates in the fusion with lysosomes to generate autolysosomes [43], [51], [52]. For this reason, we assessed the lysosome number per cell using LysoTracker Red (Fig. S2C). To measure lysosomal activity, cells were loaded with the bovine serum albumin conjugate DQ™-Red BSA, where intensely fluorescent fragments are detectable upon cleavage of the conjugate. Here, bafilomycin was used as a negative control to prevent DQ™-Red BSA cleavage via lysosomes [53]. As depicted in Fig. S2C, immE6 and immE6/E7 cells exhibited not only more lysosomes and enhanced cleavage of DQ™-Red BSA (Fig. 3C), but also a higher activity of the lysosomal enzyme cathepsin B (Fig. 3D). However, even a wide spectrum of different inhibitors of lysosomal enzymes was not able to increase the levels of pro-IL-1β in these cells (Fig. 3E). One can therefore conclude that the observed differences in lysosomal and cathepsin B activity cannot account for the low basal amounts of pro-IL-1β in immE6 and immE6/E7 cells compared to immE7 cells (Fig. 1C).

### Pro-IL-1β is ubiquitinated and degraded in a proteasome-dependent manner in HPV16-positive cells

**Figure 4:**
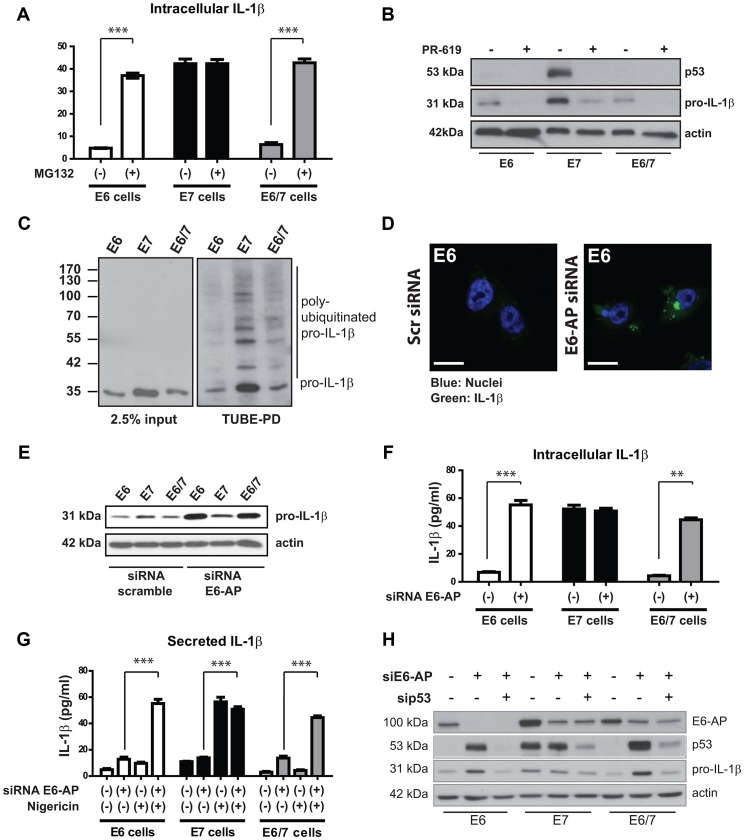
Proteasome inhibition or knock-down of E6-AP increases the levels of pro-IL-1β in immortalized E6-positive cells. A) ELISA of intracellular IL-1β from untreated immortalized HPV-positive cells and cells incubated with 10 µM of the proteasome inhibitor MG132 for 6 h. B) Western blot analysis of pro-IL1β and p53 in immortalized keratinocytes after inhibition of deubiquitinases using PR-619 (10 µM) for 6 h prior to protein extraction. C) Detection of poly-ubiquitinated pro-IL-1β in immortalized keratinocytes by Western blotting. Cells were treated with MG132 for 6 h prior to protein extraction and pull-down of ubiquitinated proteins was performed by the tandem ubiquitin-binding entities technique (TUBE-PD, right panel). Left panel: input, representing 2.5% of the total protein extract. D) Confocal microscopy analysis of IL-1β (green) in immE6 cells after knock-down of E6-AP by siRNA or scrambled siRNA delivery used as a control. Nuclei (blue) were stained using Hoechst dye solution; scale bars represent 10 µm. E) Western blot analysis of pro-IL-1β after the knock-down of E6-AP. F) ELISA of intracellular IL-1β from immortalized HPV-positive cells after the knock-down of E6-AP by siRNA (+) or control knock-down.(−) G) ELISA of IL-1β secretion from immortalized HPV-positive cells after E6-AP knock-down and/or after NALP3 inflammasome activation using 50 µM of nigericine for 6 h. H) Knock-down of p53 and/or E6-AP in immortalized cells. Cells were transfected with 30 pmoles of the respective siRNA against E6-AP or p53 and incubated for 72 h prior to protein extraction and Western blot analysis. The bar graphs shown in A (ANOVA ***p<0.05), D (ANOVA ***p<0.001, **p<0.01) F and G (ANOVA ***p<0.001) represent the mean levels (± SEM) of three independent experiments.

Having excluded autophagy and lysosomal degradation as potential mechanisms for the reduced pro-IL-1β levels in E6-positive cells (Fig. 3), we reasoned that a post-translational degradation process may account for the reduced intracellular levels of pro-IL-1β in E6-positive cells compared to immE7 cells. In cells infected with high-risk HPVs, the E6 oncogene is known to interact with the E3 ubiquitin ligase E6-AP forming a trimeric complex with p53 [54], [55]. This targets p53 for degradation through the ubiquitin-proteasome pathway, resulting in the loss of apoptotic functions and relieve of cell cycle control [54], [55]. Since E6-AP was shown to ubiquitinate a variety of other cellular proteins [56], we incubated immortalized cells for six hours with the proteasome inhibitor MG132 and subsequently measured the intracellular levels of IL-1β by ELISA. As shown in Fig. 4A, MG132 treatment increased the amount of IL-1β protein in immE6 and immE6/E7 cells to similar quantities as found in immE7 cells. Note that elevation of IL-1β on the protein level was not due to an increase of the corresponding mRNA after MG132 treatment (Fig. S3A), further supports the notion that a post-translational control mechanism is responsible for IL-1β degradation in immortalized keratinocytes.

Next, we analyzed whether IL-1β is a direct substrate for ubiquitination which ultimately targets proteins for proteasomal degradation. For this purpose, immortalized keratinocytes were treated for six hours with the deubiquitinase inhibitor PR-619 [57] (Fig. 4B). Under these conditions, both IL-1β and p53 were degraded in immE7 cells, thereby mimicking the effect of enhanced p53 and pro-IL-1β degradation observed in the presence of the E6 oncoprotein. Additionally, to directly confirm ubiquitination of pro-IL-1β, the TUBE (Tandem Ubiquitin-Binding Entities) technique was applied [58], which allows the specific pull-down of ubiquitinated proteins from cell lysates and their detection by Western blotting (Fig. 4C). Using p53 as a positive control (Fig. S4A), poly-ubiquitinated pro-IL-1β could be visualized in all cell lines, where the band intensities directly reflected the overall steady-state amount of pro-IL-1β protein in the respective samples (Fig. 4C, see also Fig. 1C for comparison).

### E6-AP and p53 are involved in the post-translational control of pro-IL-1β

To prove whether E6-AP contributed to the degradation of pro-IL1β, we used siRNA delivery to knock-down E6-AP in immE6, immE7 and immE6/E7 cells (Fig. S3B). As visualized by confocal microscopy, knock-down of E6-AP leads to an accumulation of pro-IL-1β in the cytosol of transfected cells (Fig. 4D) that could also be detected by Western blot (Fig. 4E). Measurement of intracellular IL-1β amounts by ELISA (Fig. 4F) revealed that the knock-down of E6-AP in E6-positive cells re-constituted the levels of IL-1β to a similar extend as obtained after MG132 treatment (Fig. 4A). As shown for MG132 treatment (Fig. S3A), knock-down of E6-AP had also no effect on *IL1B* transcription (Fig. S3B), again demonstrating that E6-AP depletion resulted in post-translational stabilization of pro-IL-1β.

To test whether the knock-down of E6-AP and subsequent activation of the NALP3 inflammasome leads to the secretion of mature IL-1β, cells were treated with the ionophore Nigericin [23]. As presented in Fig. 4G, Nigericin treatment alone had no significant effect on immE6 and immE6/E7 cells, but induced a strong IL-1β release in immE7 cells that was similar to that observed after adenoviral infection (Fig. 1A). Notably, in immE6 and immE6/E7 cells, delivery of siRNA against E6-AP and the subsequent treatment with Nigericin lead to a secretion of IL-1β that was comparable to that of immE7 cells (Fig. 4G). This finding demonstrated that the elevation of intracellular pro-IL-1β levels alone was sufficient to restore the secretion capacity of E6-positive cells.

Since both MG132 treatment and E6-AP knock-down also restored p53 protein, we next analyzed whether the increase in pro-IL-1β levels was a p53-dependent process. For this purpose, double knock-downs of E6-AP and p53 were performed (Fig. 4H). While E6-AP siRNA was able to strongly elevate the levels of pro-IL-1β in E6- and E6/E7 positive cells, co-delivery of siRNA directed against p53 completely abrogated this effect. In contrast, knock-down of p53 in E7 cells only lead to a minor reduction, probably resulting in the autophagosomal degradation of pro-IL-1β upon p53 inactivation [59]. One can therefore conclude that pro-IL-1β is post-translationally controlled in immE6 and immE6/E7 cells by the interplay between E6-AP, p53 and HPV16 E6. However, co-immunoprecipitation studies in HPV16-positive SiHa cells did not show a direct interaction between p53 and pro-IL-1β under our experimental conditions where p53 and E6-AP association could be detected (Fig. S4D).

### The role of E6 oncoproteins in the control of intracellular pro-IL-1β levels

**Figure 5:**
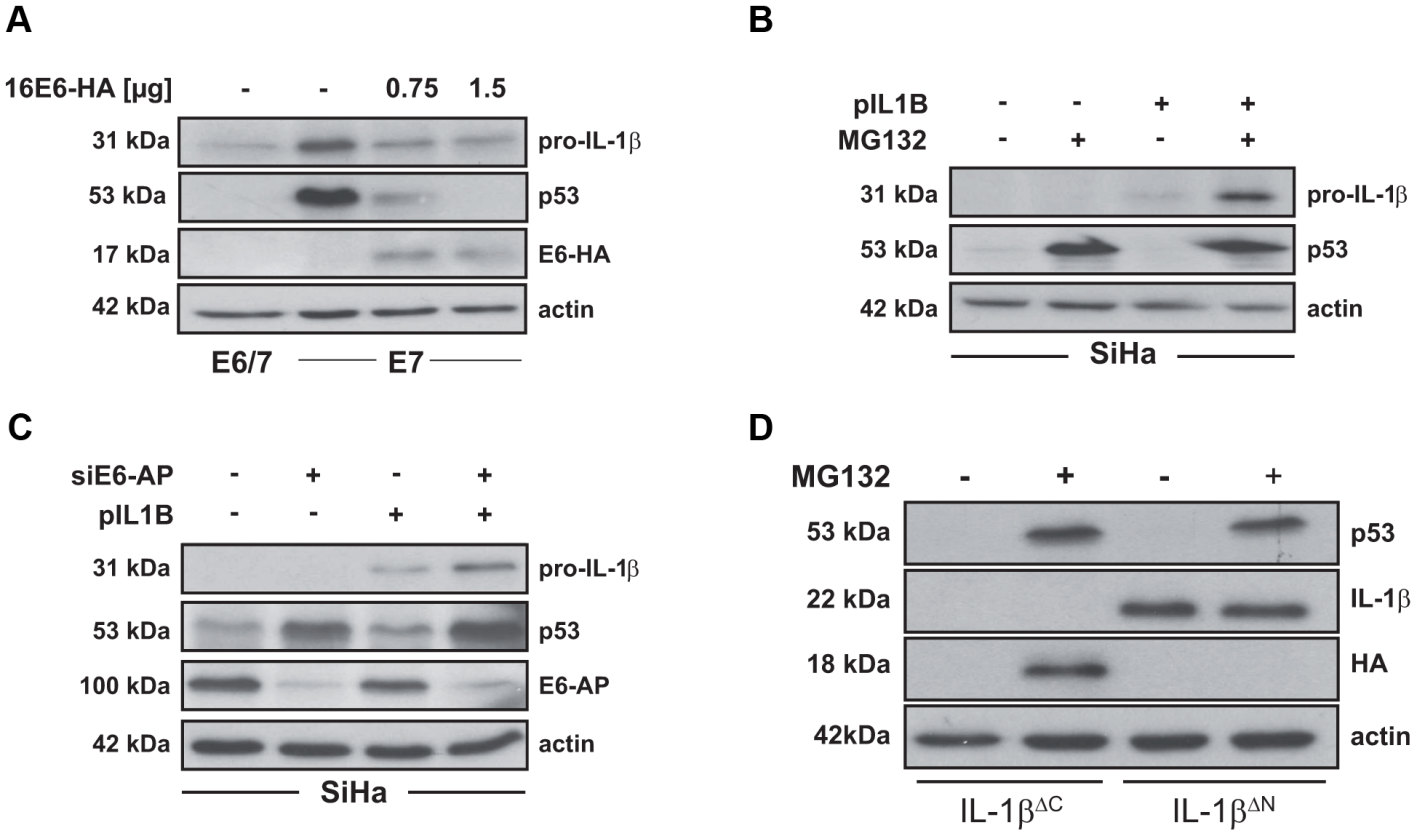
IL-1β is degraded in a proteasome-dependent manner in HPV16 E6-positive cells. A) Western blot analysis of pro-IL-1β and p53 in immE7 cells after ectopic expression of HA-tagged HPV16 E6. (−): non-transfected cells (E6/7) or cells transfected with empty vector (E7). B) Western blot analysis of pro-IL-1β and p53 levels in the HPV16-positive cervical carcinoma cell line SiHa 24 h after transfection with an IL-1β-encoding expression plasmid (pIL1B) in the presence or absence of 5 µM MG132 for 6 h. C) Western blot analysis of pro-IL-1β, p53 and E6-AP in SiHa cells after the knock-down of E6-AP by siRNA and subsequent transfection with an IL-1β expression plasmid (pIL1B). D) Western blot analysis of SiHa cells expressing truncated IL-1β. Cells were transfected with 1 µg of plasmids either lacking the C-terminus from amino acid 117 to 269 (IL-1β^ΔC^-HA) or lacking the N-terminal part from amino acid 1 to 76 (IL-1β^ΔN^) of pro-IL-1β. Cells were incubated for 18 h and treated for 6 h with MG132 (10 µM) prior to protein extraction and Western blot analysis.

Since siRNA directed against the E6 oncoprotein strongly affects the viability of the respective cell lines [60], we alternatively transfected immE7 cells with HA-tagged HPV16 E6 and analyzed the levels of pro-IL-1β by Western blot. The delivery of E6 not only reduced p53 levels in a dosage-dependent manner, but also lead to a rapid decrease of the steady state amounts of pro-IL-1β 24 hours after transfection (Fig. 5A). This suggests that the expression of E6 appears to immediately interfere with the processing and half-life of pro-IL-1β in a dominant fashion when delivered into immE7 cells. Transfection of HPV18 E6 protein into immE7 cells showed the same effect, whereas the reduction of pro-IL-1β after ectopic expression of E6 of low-risk HPV6 or HPV11 was only marginal (Fig. S4B).

Conversely, we raised the question whether the stability of ectopically expressed pro-IL-1β is also affected when introduced into SiHa cervical carcinoma cells, which lack endogenous transcription of the corresponding gene (Fig. 1D and S1C). Using increased p53 levels as an internal control for effective inhibition of the proteasome, ectopically expressed pro-IL-1β could only be stabilized in the presence of MG132, but was barely detectable when the proteasome inhibitor was omitted (Fig. 5B). Carrying out an equivalent experimental set-up but in the presence of E6-AP siRNA, the same effect was obtained (Fig. 5C). As depicted by Western blotting, the knock-down of E6-AP resulted not only in a strong restoration of endogenous p53, but also in the stabilization of ectopically expressed pro-IL-1β. These data indicate that the stability of pro-IL-1β is functionally linked to an E6-AP/p53-dependent pathway.

In order to determine which region of pro-IL-1β confers its instability in the presence of E6, we transfected SiHa cells with pro-IL-1β deletion mutants that either expressed only the N-terminus (IL-1β^ΔC^-HA) that is lacking the region corresponding to the mature form of pro-IL-1β or an N-terminally truncated version of the protein (IL-1β^ΔN^) that contains the region of the mature form. As depicted in Fig. 5D, cells that were transfected with IL-1β^ΔC^-HA did not display any protein expression unless the proteasome was inhibited. Conversely, transfection of IL-1β^ΔN^ yielded a strong signal even in untreated cells whose intensity did not increase further upon MG132 treatment. This finding demonstrates that the N-terminus of pro-IL-1β is required to target the protein for proteasomal degradation, whereas the C-terminus of IL-1β appears to be very stable in the presence of HPV16 E6.

### HPV-positive tissue samples show a decrease of pro-IL-1β with progressing cell transformation

**Figure 6:**
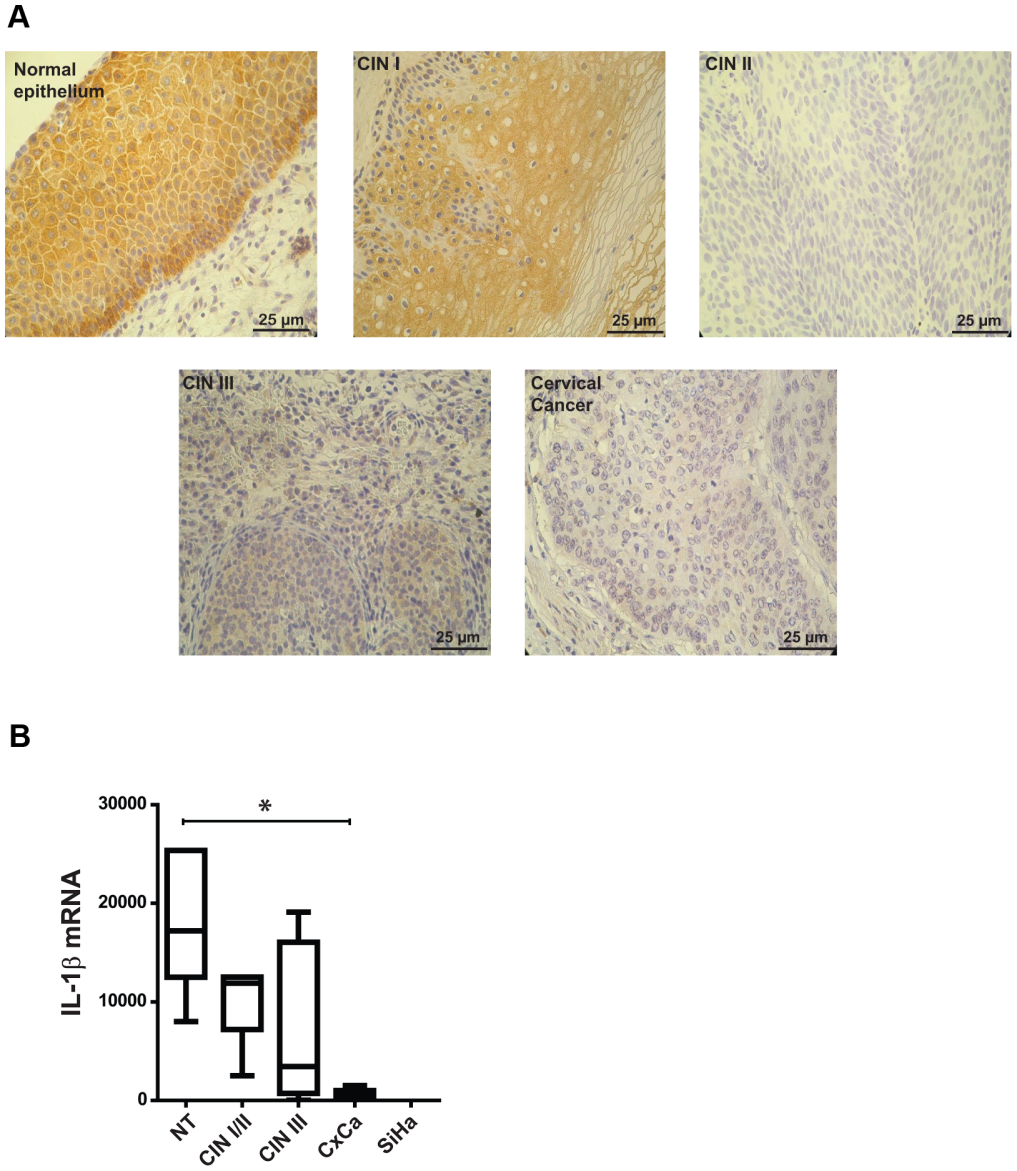
Analysis of IL-1β expression in normal and HPV16-positive cervical tissues. A) Immunohistochemical analysis of IL-1β expression in normal epithelium and HPV-positive lesions differing in their progression grades CIN I to CIN III and cervical tumors; scale bars represent 25 µm. B) qPCR analysis of IL-1β cDNA derived from samples negative for intraepithelial lesion and malignancy (NT), different HPV-positive lesions (CIN I, II, III) and cervical tumors (CxCa); ordinate: expression as fold changes using SiHa cells as reference which was arbitrarily set as 1. The pictures in A are a representative example of 25 biopsies analyzed from normal tissue (n = 5) and HPV-positive lesions of different donors (n = 25). The box-and-whisker blot in B represents the mean values from three to five samples for each group depicted in the graph (± SEM). ANOVA *p<0.01.

To confirm whether our *in vitro* data are also of clinical relevance, sections of normal cervical tissue, different progression states (cervical intraepithelial neoplasia, CIN I-III) and cervical cancer biopsies were studied by immunohistochemistry. Considering representative examples depicted in Fig. 6A, immunohistochemical examination of four out of five normal tissue samples showed ubiquitous staining for IL-1β throughout the whole section from parabasal to suprabasal cells with the most intensive signals coinciding with the basal layer. While three out of five CIN I lesions still show a positive, but diffuse staining, sections with medium and higher degree of neoplasia (CINII/III lesions) as well as samples from cervical cancer patients gradually lack detectable IL-1β expression. In parallel experiments where pro-IL-1β RNA was extracted from cervical smears (characterized by cytology and HPV DNA status) and analyzed by qPCR (expressed as box-and-whisker diagram, Fig. 6B), the same tendency towards transcriptional silencing of *IL1B* could be discerned.

Notably, low-risk types such as HPV6 obviously prefer the mode of gene suppression instead of post-translational labilization of the IL-1β precursor protein to escape immune surveillance (see Fig. S4B), since five out of five genital warts showed a complete shut-off in IL-1β transcription when compared to primary keratinocytes which were used as a positive control (Fig. S4C).

## Discussion

The innate and adaptive immune system plays a critical role in the prevention, limitation and clearance of high-risk HPV infections, which are known to be the primary cause of anogenital cancer [61]. Due to their low permissive cycle, viral burst size and the absence of a distinctive viremia, the humoral response against viral capsids is weak and can only be detected around six months after a natural infection [62], [63]. Conversely, this period of time is apparently sufficient to establish a persistent infection, thereby escaping from innate immune surveillance as a first line of antiviral defense. During this time interval, high-risk HPVs, like other potential tumor viruses, attack several hubs within the interactome of their target cell to circumvent innate immunity and prevent cell elimination [5], [8], [64]. There are several strategies how this can be mediated including targeting of cytokines [13] and chemokines [14] as well as by inhibition of antigen presentation [65]. In addition, the viral oncogenes promote cell cycle progression [66] and therefore favor the accumulation of forthcoming premalignant cells that are prone to convert towards malignancy [1], [66].

Beside type I interferons [67], pro-inflammatory cytokines such as IL-1β are also crucial effector molecules that are required to mount a proper innate immune response against viral infections [27]. In view of its strong immune stimulatory effects and its pleiotropic function, IL-1β release is tightly controlled at multiple levels, such as transcription, post-translational maturation in an inflammasome/caspase-1-dependent manner and secretion of the biologically mature protein [20], [21], [27], [28], [36], [68].

In the present report we show that intracellular stability of pro-IL-1β is impaired by a post-translational control mechanism in HPV16-immortalized human keratinocytes (Fig. 4), therefore limiting their capacity to release mature IL-1β upon infection with adenovirus (Fig. 1A) or after treatment with Nigericine (Fig. 4G) which activates the NALP3 inflammasome.

Similar to our previous studies on the regulation of the keratinocyte-specific interferon-κ [13], the E6 oncoprotein is playing a central role in this process, since the intracellular levels of pro-IL-1β (Fig. 1B), its secretion as mature IL-1β (Fig. 1A) and its biological activity were not impaired in immE7 cells (Fig. S1B). Moreover, abrogation of IL-1β availability during HPV-induced carcinogenesis seems to be a two-step mechanism, since cervical carcinoma cells (Fig. 1D) as well as human cervical tumor tissues (Fig. 6B) showed either a strongly reduced or even an absence of the corresponding mRNA. The observed higher levels of IL-1β mRNA in immortalized cells in contrast to primary keratinocytes (Fig. 1D) could be due to a general viral oncogene-mediated increase of NF-κB signaling [69] that may activate the gene itself, which is known to be NF-κB responsive [70]. Nonetheless, the impairment of IL-1β release in immortalized cells and its transcriptional inactivation in malignant cells and genital warts (Fig. S4B) is obviously advantageous, since it also interrupts the well-known regulatory circuit in stimulating the expression of MCP-1 [71], [72], a chemokine, attracting cells of the monocyte/macrophage lineage and activating them to release growth-inhibitory cytokines [73], [74]. Consistent with this notion is also a growth retarding effect on cervical carcinoma cells upon heterotransplantation into nude mice when IL-1β was ectopically expressed [75].

To get insight into the molecular pathway by which IL-1β is regulated in HPV-positive cells, we investigated whether modulation of the inflammasome may account for the decreased IL-1β levels in E6-positive cells. Examples in which way this can be mediated have been reported for several DNA and RNA viruses such as herpesviruses that interfere with the assembly of the inflammasome, influenza viruses encoding a caspase-1 specific inhibitor and orthopoxviruses that secrete an IL-1β binding protein that acts as a decoy receptor [27]. Pro-IL-1β is processed to its mature form by caspase-1 after NALP3 activation and the subsequent autoproteolytic cleavage of pro-caspase-1 [21]. Studies in caspase-1 knock-out mice have shown that these animals have an increased susceptibility to a variety of infections, because IL-1β maturation is impaired [17]. Conversely, caspase-1 seems not to be involved in the immune surveillance against *Chlamydia trachomatis* [76], since its activity is even required for efficient and optimal growth of chlamydial inclusions in cervical epithelial cells [77] This aspect is interesting especially in the context of an epidemiologically significant association between *Chlamydia* infection, persisting HPV and the development of cervical cancer [78]. Hence, our finding that a similar caspase-1 activity can be observed within all immortalized cells independent of which viral oncoprotein is expressed (Fig. 2B and C) may provide an explanation for a selective benefit of such a co-habitation in maintaining caspase-1 activity, but shutting down IL-1β secretion also in infected individuals.

In attempts to identify the mechanism involved in pro-IL-1β regulation, we also investigated autophagy-related processes in our cell system (Fig. 3A and B). Autophagy is considered to be an evolutionary highly conserved catabolic pathway, targeting proteins or cell organelles for lysosomal degradation. Moreover, autophagy is also interconnected to many intracellular processes such as energy sensing, apoptosis and inflammasome signaling [20], [53]. Nonetheless, despite increased basal levels of LC3 punctae in autophagosomes (Fig. S2A) and enhanced lysosomal activity in immE6 and immE6/E7 keratinocytes (Fig. 3C), inhibition of autophagy (Fig. 3A and B) or treatment with various inhibitors of lysosomal proteases failed to increase intracellular pro-IL-1β levels (Fig. 3E).

On the other hand, incubation of immE6 and immE6/E7 cells in the presence of MG132 could elevate intracellular pro-IL-1β protein to levels comparable to immE7 keratinocytes (Fig. 4A). Indeed, precedential cases have been reported in a human monocytic cell line (THP-1) or in murine bone marrow-derived macrophages where the proteasome inhibitor MG132 was also capable to significantly raise the intracellular half-life of pro-IL-1β [79], [80]. This indicates that the intracellular amounts of pro-IL-1β can also be regulated by proteasomal degradation in a NALP3-independent manner [17].

The ubiquitin proteasome pathway targets cellular proteins for proteasomal degradation by site-specific poly-ubiquitination of lysine residues [81]. This process is reversible and balanced by a tightly controlled interplay between ubiquitin ligases and deubiquitinating enzymes [82], [83]. The treatment with PR-619, a non-selective reversible inhibitor of deubiquitinases, lead to a strong reduction of both pro-IL-1β and p53 levels in immortalized keratinocytes, showing common pathways controlling their intracellular half-life (Fig. 4B). Similar to p53, poly-ubiquitinated pro-IL-1β could be visualized after MG132 treatment (Fig. 4C), confirming a proteasome-dependent degradation process that is increased in the presence of the E6 oncogene.

In cells infected with high-risk HPVs, the viral oncoprotein E6 targets the E3 ubiquitin ligase E6-AP, forming a trimeric complex with p53 and facilitating its ubiquitination and subsequent degradation via the ubiquitin proteasome pathway [55]. Notably, E6-AP also has an additional function, being a transcriptional co-activator of steroid hormone receptor [84]. This is important in the context of HPV-induced carcinogenesis, since the long-term use of oral contraceptives significantly increases the risk for the development of cervical cancer in HPV-positive women [85], [86]. To distinguish whether the increased amounts of pro-IL-1β after MG132 treatment were due to a direct involvement of E6-AP or simply due to an extended half-life of a labile protein that controls pro-IL-1β stability, siRNA experiments were performed. As shown by confocal microscopy (Fig. 4D), Western blot (Fig. 4E) and ELISA (Fig. 4F), the specific knock-down of E6-AP restored intracellular pro-IL-1β levels and even rescued the secretion of mature IL-1β after NALP3 inflammasome stimulation (Fig. 4G). Remarkably, this process is directly depending on the availability of p53, because its simultaneous knock-down counteracts intracellular pro-IL-1β accumulation, as achieved by preceding inactivation of E6-AP alone (Fig. 4H). Since neither the treatment with MG132 nor the knock-down of E6-AP lead to an increase in IL-1β mRNA levels (Fig. S3A and B), a role of p53 as a transcriptional activator of the *IL1B* gene can be excluded. Although co-immunoprecipitation experiments demonstrate not direct binding between p53 and pro-IL-1β, under our experimental conditions where E6-AP and p53 interact (Fig. S4A and D), the latter seems to be involved in the half-life control of pro-IL-1β. Hence, mass spectrometric analyses [87] should identify accessory proteins of pro-IL-1β that modulate its intracellular stability in high-risk HPV-positive cells. Nevertheless, since p53 can be also considered as a central hub within the cellular network, its inactivation by E6 and the successive downstream effects also on the keratinocyte-specific interferon-κ and chemokines such as MCP-1 [14] allows the virus to efficiently circumvent essential components of the innate immunity in a one-step mode by reducing p53 availability.

These findings identify a novel role of p53 and E6-AP within the cellular interactome, which apart from inactivating p53 [55] or proteins involved in cell cycle control or differentiation [56], also controls the levels of IL-1β, a central cytokine that is necessary to orchestrate a potent innate immune response [20], [28]. Moreover, the importance of E6-AP in the development of HPV-induced carcinomas has been elegantly shown in an HPV16-transgenic mouse model after cross-breeding with E6-AP null animals where the loss of E6-AP completely abrogated the E6-induced development of cervical cancer upon chronic estrogen treatment [88].

In order to study the role of E6 in greater detail, transfection of HA-tagged HPV16 E6 expression vectors into E7-positive keratinocytes strongly down-regulated the levels of both pro-IL-1β and p53 in a dose-dependent manner (Fig. 5A). The same effect could be noted in immE7 cells that expressed the E6 proteins of HPV18, while E6 of the low-risk types (HPV6 or HPV11) had only a minor influence on pro-IL-1β stability (Fig. S4B). In contrast, monitoring the levels of p53 after HPV6 and HPV11 E6 expression in comparison to empty vector transfected E7-positive keratinocytes as reference, a discernible effect could be observed (Fig. S4B). While some studies claimed that E6 proteins from low-risk HPV are neither able to bind nor degrade p53 [54], [89], [90], other groups showed the opposite, although with weaker efficiency than high-risk HPV E6 [91]–[93]. Discrepancies like this may be explained either by the usage of different cell lines, the respective gene dosage or the techniques to monitor the final read-out. For our transfection experiments we used human keratinocytes as recipients which are the natural target cells for an HPV infection. One possible explanation for the decrease of p53 in our experiments could be that the presence of HPV16 E7 is somehow predisposing p53 for reduction of its half-life upon low-risk HPV E6 delivery, since E7 can up-regulate SIRT, an aging-related NAD-dependent deacetylase. This can lead to p53 deacetylation [94] thereby inherently affecting its stability [95].

As shown in Fig. 5B and C, ectopic expression of pro-IL-1β in combination with MG132 treatment or E6-AP siRNA knock-down was able to stabilize pro-IL-1β in cervical carcinoma cells. This indicates that even though the endogenous gene is no longer transcribed, the post-translational process that controls the half-life of pro-IL-1β is still active in the presence of E6. One can therefore propose that there exists a two-step silencing mechanism of IL-1β towards cervical cancer: i) on the post-translational level during immortalization and ii) transcriptionally by gene silencing in malignant cells. Since the absence of transcription was also noted in the HPV-negative C-33 A cell line (Fig. S1C), U2OS, HEK 293 and H1299 cells (data not shown), this second step of IL-1β inactivation is likely not HPV-dependent, but might merely reflect a selective growth advantage in different types of tumors.

The fact that E6-positive SiHa cells still maintained the E6-mediated post-translational degradation of ectopically expressed pro-IL-1β allowed us to assess the region of pro-IL-1β that confers its instability in these cells. Different *in vitro* studies provided some hints that mature IL-1β is less susceptible to various proteases than its pro-form [96]. To characterize the region that is responsible for degradation, N- or C-terminally truncated mutants of pro-IL-1β were expressed in SiHa cells (Fig. 5D). As already anticipated the deletion mutant containing amino acids 1-116 of pro-IL-1β was highly susceptible to proteasomal degradation and could only be stabilized by MG132 treatment, whereas the N-terminally truncated mutant protein (lacking amino acids 1-76 and containing the full-length region of mature IL-1β) was readily expressed even in untreated cells. We are currently analyzing whether one or more of the five lysine residues within the N-terminal pro-protein region of IL-1β are responsible for its increased degradation in the presence of E6.

Finally, to estimate the clinical relevance of our *in vitro* data, initial validation of human biopsy material shows a progressive loss of IL-1β, as monitored both by immunohistochemical staining (Fig. 6A) and quantitative RT-PCR of tissue samples (Fig. 6B), representing different grades of intraepithelial neoplasia (CIN I–III) and cervical cancer. We currently do not know at which time there is a switch from post-translational labilization of pro-IL-1β towards silencing of the gene itself during multi-step progression to cervical cancer. However, our recent studies indicate that the *IL-1B* gene gets converted into heterochromatin, a situation already described for the keratinocyte-specific interferon-κ [13].

Notably, monitoring IL-1β transcription in genital warts infected by HPV6, the same situation could be discerned (Fig. S4C). Persistent genital warts are characterized by a lack of infiltrated immune cells where low numbers of intraepithelial CD8^+^ T cells and mononuclear cells are present mainly in the stroma [10]. Since IL-1β mediates the migration of leukocytes and promotes the activation of T cells [18], its consecutive transcriptional inactivation apparently provides a selective advantage also for low-risk HPV-infected cells to escape immune surveillance, since their ability to labilize IL-1β in a post-translational manner is only marginal when compared to HPV16 or HPV18 E6 (Fig. S4B). A broader study of tissue specimen will answer the question whether the status of IL-1β expression can also be used as an additional marker for progressing lesions.

Taken together, our data reveal an effective and novel mechanism how high-risk HPV circumvent the function of IL-1β. As a consequence, attenuated innate immune response against HPV-infected cells may favour viral persistence, which is an important step in the initiation of cellular transformation and tumorigenesis. A detailed understanding of how high- risk HPV E6 can interfere with the expression and maturation of different pro-inflammatory cytokines, such as IL-1β via E6/E6-AP/p53 interaction or via *de novo* methylation as recently shown for the keratinocyte-specific type I interferon IFN-κ [13], should allow the development of new strategies to treat existing HPV lesions before their progression to invasive tumors.

## Materials and Methods

### Ethics statement

Samples were donated from HPV infected patients or health donors using protocols approved by the Charité Campus Benjamin Franklin, Berlin, Germany and the Deutsche Klinik Bad Münder, Hannover, Germany. Subsequently the samples were analysed anonymously where informed consent was not required.

### Cell culture

HPV16-immortalized keratinocytes expressing the E6 and/or E7 oncogene [97] were cultivated in Keratinocyte-SFM medium containing rhEGF and bovine pituitary gland extract (Life Technologies). Tumor cell lines CaSki, SiHa, HeLa and C-33 A were grown in Dulbecco's Modified Eagle Medium (Sigma) containing 10% fetal calf serum (Linaris). Human Umbilical Vein Endothelial Cells (HUVEC) (EMD Millipore) were cultivated in EndoGRO-Low Serum complete medium (EMD Millipore). Human neonatal Epidermal Keratinocytes (Life Technologies) were cultivated in EpiLife medium (Life Technologies) containing 60 µM calcium supplemented with Human Keratinocyte Growth Supplement (Life Technologies).

### GFP-adenovirus infection

Infection using GFP-adenovirus 5 (Vector Biolabs) was carried out as described previously [98]. Briefly, 100 MOI of virus was added to the primary keratinocytes and HPV16-positive cells in the respective medium and incubated for 1 h. The cells were washed twice with phosphate buffered saline and cultivated again for 24 h. Subsequently the supernatants were collected and stored at −80°C until used to determine the amount of secreted IL-1β by ELISA.

### ELISA

ELISAs were performed using the Human IL-1 beta ELISA Ready-SET-Go! Kit (eBioscience) according to the manufacturer's instructions. For intracellular protein analyses, 15–30 µg of total protein was applied in triplicates to the coated plate. Secreted IL-1β levels were measured by applying 100 µl of supernatants of the respective medium to the coated ELISA plate.

### Protein extraction and Western blotting

After treatments or transfections, ∼1×10^6^ cells were collected, washed in 1×PBS and resuspended in RIPA buffer (20 mM Tris pH 7.5; 150 mM NaCl; 1 mM Na_2_EDTA; 1 mM EGTA; 1% NP-40; 1% sodium deoxycholate) including 1× complete protease inhibitor cocktail (Roche). Samples were incubated for 30 min on ice and subsequently centrifuged for 30 min at 4°C and 13,000 rpm. The supernatants were quantified using Bio-Rad Protein Assay Dye Reagent Concentrate (Bio-Rad). 50–80 µg of denatured proteins was used for Western blotting. After transfer, the filters were incubated with the following antibodies: anti-human interleukin-1β (IL-1b-I), 3415-3-250, (MABTECH), anti-p53 antibody (DO-1), sc-126 (Santa Cruz Biotechnology), anti-E6AP antibody (H-182), sc-25509 (Santa Cruz Biotechnology), anti-actin clone C4 (MP Biomedical), anti-LC3A D50G8 (Cell Signalling), rat anti-HA clone 3F10 (Roche).

### Immunoprecipitation

After transfections or treatments, ∼1×10^6^ cells were collected, washed in 1×PBS and resuspended in non-denaturing lysis buffer (20 mM HEPES, 0.15 M NaCl, 5 mM EDTA, 10% Glycerol, 0.5% Triton X-100) including 1× complete protease inhibitor cocktail (Roche). Samples were incubated for 30 min on ice and subsequently centrifuged for 30 min at 4°C and 13,000 rpm. The supernatants were quantified using Bio-Rad Protein Assay Dye Reagent Concentrate (Bio-Rad) and 500 µg of protein was used for immunoprecipitation. Non-specific binding in the cell extracts was removed by adding 50 µl of protein G–Sepharose beads (Santa Cruz Biotechnology) equilibrated with non-denaturing lysis buffer and rocking for 1 h at 4°C. After removing the beads, the cell extract was incubated with 5 µg of anti-p53 monoclonal antibody (DO-1) or 5 µg of normal mouse IgG sc-2025 (Santa Cruz Biotechnology) used as control for 6 h at 4°C. Then, 10 µl of protein G–Sepharose beads equilibrated with non-denaturing lysis buffer was added to each reaction mixture and rocked for 12 h at 4°C. The beads were washed five times with non-denaturing lysis buffer and analyzed by Western blotting using interleukin-1β (IL-1b-I), anti-p53 antibody (DO-1) and anti-E6AP antibody (H-182). Additional co-immunoprecipitation experiments were performed using 4 µg of interleukin-1β antibody or 0.8 µg of GFP antibody (Roche) for immunoprecipitation. Western blot analyses employed the previously described antibodies according to [99]. HA-tagged pro-IL-1β was detected with an antibody directed against the HA-tag (Roche).

### RNA extraction and reverse transcription

RNA was extracted from cells using the RNeasy Mini Kit (Qiagen) according to the manufacturer's instructions. RNA concentrations were determined photometrically and 1 µg of RNA was reverse transcribed using RevertAid Reverse Transcriptase (Fermentas) and dT_22_ primers according to the manufacturer's protocol. A 1∶5 dilution of the resulting cDNA was used for semi-quantitative and quantitative PCR analyses.

### Semi-quantitative and quantitative PCR analyses

Semi-quantitative RT-PCRs were performed by using DreamTaq Green DNA polymerase (Fermentas) according to the manufacturer's instructions. The following primers were used: GAPDH forward: 5′-GCCTTCCGTGTCCCCACTGC-3′, GAPDH reverse: 5′-GCTCTTGCTGGGGCTGGTGG-3′, Caspase-1 forward: 5′-TCTTCCTTTCCAGCTCCTCA-3′, Caspase-1 reverse: 5′-CGCTGTACCCCAGATTTTGT-3′, IL-1β forward: 5′- GGGCCTCAAGGAAAAGAATC-3′, IL-1β reverse: 5′-AGCTGACTGTCCTGGCTGAT-3′, IL-6 forward: 5′-TCGAGCCCACCGGGAACGAA-3′, IL-6 reverse: 5′-GCAGGGAAGGCAGCAGGCAA-3′, CCL-20 forward: 5′-GGCGAATCAGAAGCAAGC-3′, CCL-20 reverse: 5′- TTCCATTCCAGAAAAGCCAC-3′, E6-AP forward: 5′-GCGGGGGCGACGACAGGTTA-3′, E6-AP reverse: 5′-TGCAGCTTCTCCATCCTGCAAGC-3′. Quantitative real-time PCR (qPCR) was performed with an ABI 7300 qPCR cycler (Applied Biosystems) using Maxima SYBR Green/ROX qPCR Master Mix (Fermentas) and the following primers: IL-1β forward: 5′-AGGCACAAGGCACAACAGGCT-3′, IL-1β reverse: 5′-GGTCCTGGAAGGAGCACTTCAT-CTG-3′, IL-1α forward: 5′-TGGTAGTAGCAACCAACGGGA-3′, IL-1α reverse: 5′-ACTT-GATTGAGGGCGTCATTC-3′, IL-18 forward: 5′-ATCGCTTCCTCTCGCAACAA-3′, IL-18 reverse: 5′-TCCAGGTTTTCATCATCTTCAGC-3′, IL-33 forward: 5′-TAGGAGAGAAACC-ACCAAAAGG-3′, IL-33 reverse: 5′-ACTTTCATCCTCCAAAGCAAAAGT-3′. As a normalization control, TBP ( = TATA-Box binding protein)-specific primers were used. TBP forward: 5′-GAGTCGCCCTCCGACAAAG-3′ and TBP reverse: 5′-GTTTCCTCTGGGATTCCATCG-3′.

### Measurement of Caspase-1 activity and Caspase-1 inhibition

Caspase-1 activity assay was performed by staining the cells for 4 h at 37°C with 20 µM of the cell-permeant R110-YVAD (Rhodamine 110-Based Caspase-1 substrates; Life Technologies). The fluorescence emission of caspase-1 (Excitation: 485 nm/Emission: 528 nm) were read in a Synergy 2 multireader (BioTek) without fixation. For the pharmacological inhibition of caspase-1, 250 nM of the caspase-1 inhibitor I cell permeable (Calbiochem) for 5 h was used.

### Transduction of immortalized keratinocytes with GFP-LC3 Lentivirus

The GFP-LC3 expressing lentivirus was a kind gift from Dr. Chiramel and Dr. Bartenschlager from Heidelberg University. Shortly, the lentiviral particles were mixed with Polybrene 5 µg/ml (Sigma) and incubated with the immortalized keratinocytes for 6 h and then replaced by normal culture medium. 72 h after infection, the target cells were seeded in 6 well plates and selection with puromycin (2 to 3 µg/ml) was performed for 12 days. The pooled puromycin resistant clones were tested by fluorescence microscopy and Western blot analysis.

### Inhibitors, quantification of lysosomes, cathepsin B activity and autophagy

Quantification of lysosomes was performed using LysoTracker Red and lysosome activity with DQ Red BSA (Life Technologies). Cathepsin B activity was monitored by incubating the cells with Magic Red (Immunochemistry technologies) according to the manufacturer's instructions. Inhibitors of autophagy used: 1 mM 3-methyladenine (Enzo Life Sciences) incubated for 8 h and 100 nM bafilomycin (Enzo Life Sciences) incubated for 8 h. Inhibitors of lysosome/protease activity: 20 µM of Calpain inhibitor PD 150,606 (Adipogen), 25 µM of cathepsin B inhibitor CA-074 (Enzo Life Sciences), 10 µM of Leupeptin (Biomol), 20 µM Vincristine (Enzo Life Sciences). All treatments were carried out for 6 h. Starvation was done by cultivating the cells in HEPES buffered saline solution (HBSS) for 8 h. For proteasomal inhibition cells were treated with 5 µM of the proteasome inhibitor MG132 (Calbiochem) for 6 h. Quantification of autophagy, lysosome amount and activity as well as cathepsin B activity were performed using high throughput high resolution fluorescence microscopy analysis (BD pathway, Beckton Dickenson) using the filter set Ex: 516 nm/Em: 590 nm. The images were analyzed using the cell imaging analysis program (CellProfiler). For inflammasome stimulation, the cells were incubated for 6 h with 50 µM of Nigericin (Enzo Life Sciences). Inhibition of deubiquitinases was carried out by incubating cells with 10 µM of PR-619 (Life sensors) for 6 h.

### Expression plasmids and transfection of cells

The complete pro-IL-1β protein coding region including the stop codon was amplified from an IL-1β open reading frame “gateway” clone (Core facility, DKFZ) with Phusion polymerase (Finnzymes) using the following primers: pIL-1β forward: 5′-CTCGAGGCCGC-CATGGCAGAAG-3′ and pIL-1β reverse: 5′-CTCGAGTTAGGAAGACACAAATTGCATG-3′. HA-tagged IL-1β was generated using the pIL-1β forward primer with the pIL-1β-HA reverse:5′-GAATTCGAAGACACAAATTGC-3′. Truncated IL-1β expression plasmids either lacking the C-terminus from amino acid 117 to 269 (IL-1β^ΔC^-HA) or lacking the N-terminal part from amino acid 1 to 76 (IL-1β^ΔN^) were constructed using the following primers: IL-1β^ΔC^ forward 5′-CTCGAGGCCGC-CATGGCAGAAG-3′, IL-1β^ΔC^ reverse: 5′-TCTCGAATTCGCATCGTGCACATAAGCC-3′ or IL-1β^ΔN^ forward: 5′-GAGACTCGAGGCCATGCTGGTTCCCTGC-3′, IL-1β^ΔN^ reverse, 5′-CTCGAGTTAGGAAGACACAAATTGCATG-3′, respectively. Amplified DNA was cloned into the pPK-CMV-E3 expression vector, where the fragments are inserted to a HA (hemagglutinin) fusion tag (PromoKine). pPK-CMV-E3 was also used to express the full length E6 open reading frame of the different HPV types where the following primers were used: HPV6E6 forward: 5′-GAGACTCGAGGCCGCCATGGAAAGTGC-3′, HPV6E6 reverse: 5′-TCTCGGATCCGGGTAACATGTCTTC-3′, HPV11E6 forward: 5′- GAGACTCGAGGCCGCCATGGAAAGTAAAG-3′, HPV11E6 reverse: 5′-TCTCGGATC-CGGGTAACAAGTCTTC-3′, HPV18E6 forward: 5′-GAGACTCGAGGCCGCCATGGCGC-GCTTTGAG-3′, HPV18E6 reverse: 5′-TCTCGAATTCAGTACTTGTGTTTC-3′. HPV16E6 in pPK-CMV-E3 and p53-YFP were provided by Dr. Dorothea Muschik.

Cervical carcinoma cells were transfected with expression plasmids using TurboFect Transfection Reagent (Fermentas), while immortalized keratinocytes were transfected using Lipofectamine 2000 Transfection Reagent (Life Technologies) according to the manufacturers' instructions.

### siRNA transfection

E6-AP or p53 knock-down was performed by transfection of Silencer Select siRNA directed against human UBE3A (S14604) or human TP53 (S605) (both Ambion) using Lipofectamine 2000 Transfection Reagent (Life Technologies) according to the manufacturer's instructions. Cells were incubated for 24 h and then transfected a second time under the same conditions. Cells were harvested after 48 h for RNA, or after 72 h for protein extraction, respectively.

### Immunofluorescence of IL-1β

After different incubation conditions, immortalized keratinocytes were fixed in 4% paraformaldehyde in PBS for 20 min at 25°C and washed for 5 min in 0.1 M glycine prior to permeabilization with 0.25% Triton X-100 in PBS for 15 min. For immunostaining, cells were incubated with the anti-human interleukin-1β (IL-1b-I) antibody (MABTECH 3415-3-250) diluted 1∶1000. The primary antibody was visualized after washing with PBS and subsequent incubation using the following secondary antibodies: rabbit anti-mouse IgG-Alexa-488 (cat: A11029) or IgG-Alexa-633 (cat: A21052; Invitrogen) for 1 h. Nuclei were stained by Hoechst solution in PBS (1∶10000; Sigma Chemicals). Fluorescence signals were obtained with a Zeiss Confocal Laser Scan Microscope (Zeiss) and the images were analyzed using the Zen imagine program (Zeiss).

### Isolation of ubiquitinated proteins

Ubiquitinated proteins were isolated from cells using the agarose-conjugated tandem ubiquitin-binding entities (TUBEs) technique (Life Sensors) according to the manufacturer's instructions. Briefly, MG132-treated keratinocytes (5×10^6^–1×10^7^ cells) were lysed in TUBE lysis buffer (50 mM Tris-HCl, pH 7.5; 0.15 M NaCl; 1 mM EDTA; 1% NP-40; 10% glycerol) containing 1× complete protease inhibitor cocktail (Roche), 20 µM MG132 and 50 µM PR-619 and were pre-cleared using protein A/G Plus Agarose beads (Santa Cruz Biotechnology). 2 mg of pre-cleared cell lysates were incubated with 30 µl of equilibrated agarose-conjugated TUBEs for 4 h at 4°C on a rotating platform, washed three times in 1×TBST and eluted by incubation at 95°C for 5 minutes in 1×SDS loading dye. Eluates were subjected to SDS-PAGE on 10% acrylamide gels and analyzed by Western blotting

### Characterization of clinical samples

Cervical smear (n = 8) fixed in PreservCyt medium (Becton Dickinson) at 4°C were collected in a routine colposcopy clinic in Bad Münder, Germany. All samples were HPV16 DNA-positive identified both by the BSGP5+/6+-PCR/Multiplex HPV Genotyping (MPG) assay that homogenously amplifies all known genital HPV types generating biotinylated amplimers of ∼150 bp from the L1 region [100] and a MPG assay with bead-based xMAP Luminex suspension array technology, which is able to simultaneously detect 51 HPV types and the β-globin gene [101], [102]. Colposcopy directed biopsies were taken from all 8 patients and histologically defined as cervical intraepithelial neoplasia (CIN1) (n = 1), CIN2 (n = 2) and CIN3 (n = 5). Furthermore, cervical cancer (CxCa) samples (n = 3) and samples negative for intraepithelial lesion and malignancy (Nil/M) (n = 5) were added from a population-based HPV prevalence study conducted in Ulaanbaatar, Mongolia in 2005 [103]. The 3 CxCa samples were well characterized by histology, the presence of HPV DNA and E6 transcription [104].

Genital benign warts (n = 5) were collected and transported in RNAlater RNA Stabilization Reagent at room temperature in a routine gynecological examination at the Bad Münder clinic, Germany and tested for HPV6 and HPV11 mRNA positivity by qPCR analysis using the following primers: HPV6-E7 forward: 5′-TTCGACTGGTTGTGCAGTGT-3′, HPV6-E7 reverse 5′-GCGCAGATGGGA-CACACTAT-3′, HPV11-E7 forward 5′-GACCCTGTAGGGTTACATTGC-3′, HPV11-E7 reverse 5′-AGTGTGCCCAGCAAAAGGTC-3′.

### RNA extraction from clinical specimens

RNA was extracted from 16 cervical smears obtained from Germany and Mongolia using the MagNA Pure 96 device (Roche Applied Science, Germany) according to the manufacturer's instructions. Briefly, 4 ml PreservCyt volume was centrifuged for 10 min at 4,700 rpm. The pellet was resuspended in 200 µl of buffer and processed into the MagNA Pure 96 system; the RNA was eluted in 50 µl of elution buffer and stored at −70°C until use. Concentration of the RNA was measured using a NanoDrop 2000. RNA from 5 genital warts was extracted cells using the RNeasy Mini Kit (Qiagen) as describe previously.

### Immunohistochemistry of IL-1β

A total of 25 cases, including normal cervical epithelium, CIN I, CIN II, CIN III and cervical cancer (5 cases of each grade) were analyzed. Formalin-fixed, paraffin-embedded sections (4 µm thick) were mounted on super-frost slides, dewaxed with xylene and gradually rehydrated. The sections were boiled for 20 min in Target Retrieval Solution (DAKO). Activity of endogenous peroxidase was blocked by 5 min incubation in Peroxidase-Blocking Solution (DAKO). The sections were then exposed to normal goat serum (Invitrogen) for 20 min. Immunohistochemical reactions were performed using a two-step staining technique (DAKO Envision system-HRP (DAB)). The samples were first incubated with the anti-human interleukin-1β (IL-1b-I), 3415-3-250, (MABTECH) diluted 1∶100 for 2 h and subsequently with the corresponding secondary antibody for 30 min at room temperature. All sections were stained with DAB (DAKO) for 10 min and counterstained with Meyer's hematoxylin. In every case, control reactions were included in which the specific antibody was substituted by the primary mouse negative control (DAKO).

### Statistical analysis

To evaluate the statistical differences between analyzed groups in figure 6, a two-sided unpaired t-test was applied. The statistical differences between analyzed groups in figure 3 were assesses by an ANOVA test.

### Databases

The following human proteins are available in the “Swiss-Prot” database (http://www.uniprot.org): IL-1β (P01584), Caspase-1 (P29466), TBP-1 (P20226), E6-AP (Q05086), p53 (P04637), actin (P60709) and GAPDH (P04406).

## Supporting Information

Figure S1 A) Control of adenoviral infection efficiency in PK, HPV16- immortalized human keratinocytes and cervical carcinoma cells 24 h after infection with 100 MOI of Ad5-GFP (white spots) at a magnification of 20×. B) RT-PCR analysis of CCL-20, IL-6 and GAPDH mRNA levels in HUVEC cells stimulated for 12 h with supernatants obtained from HPV16-immortalized cells (E6, E7, E6/7) and tumor cell lines (CaSki and SiHa) after adenoviral infection or from uninfected control cells. C) RT-PCR analysis of IL-1β mRNA levels in different cervical tumor cell lines. GAPDH served as a control gene. D) qPCR of IL-1α, IL-18 and IL-33 cDNA obtained from primary keratinocytes (PK), HPV16 immortalized keratinocytes (E6, E7, E6/7) and HPV-positive cervical carcinoma cells (Ordinate: expressed as fold changes using PK as reference which was arbitrarily set as 1).
(EPS)

Figure S2 A) Quantification of the mean fluorescence intensity (MFI) of the GFP-LC3 punctae in GFP-LC3-transduced immortalized keratinocytes cultured under normal conditions or after 8 h treatment with 100 nM of bafilomycin (to induce the accumulation of the GFP-LC3 punctae by inhibition of autophagolysosome maturation) B) Western blot analysis of pro-IL-1β and LC3A (LC3A-I and -II) in GFP-LC3-transduced cells cultured in normal versus 100 nM bafilomycin-containing medium or under starving conditions (to induce autophagy) with or without bafilomycin treatment for 8 h using HEPES buffered saline solution (HBSS). C) Quantification of the lysosome amounts (red) in cells stained for 30 min with the specific lysosomal dye LysoTracker Red (DND-99). Nuclei (blue) were stained with Hoechst dye; scale bars represent 10 µm. The analyses in A and C were performed using high throughput high resolution fluorescent microscopy analysis (BD pathway) in combination with a cell imaging analysis program (CellProfiler) in immortalized keratinocytes. The graphs in A and C show mean levels of five individual experiments performed with 10.000 events/well per experiment (± SEM) ANOVA ***p<0.001.
(EPS)

Figure S3 A) RT-PCR analysis of IL-1β mRNA levels in HPV-16-positive cells upon treatment with MG132. Cells were incubated with 10 µM MG132 for 6 h prior to RNA extraction. GAPDH levels were analyzed as an internal control. B) RT-PCR analysis of E6-AP and IL-1β mRNA levels 72 h after E6-AP siRNA delivery in immortalized keratinocytes. GAPDH was used as an internal control.
(EPS)

Figure S4 A) TUBE pull-down of ubiquitinated p53 from immortalized keratinocytes. Cells were treated with 10 µM MG132 for 6 h prior to extraction and pull-down of ubiquitinated proteins which was performed using the tandem ubiquitin-binding entities technique (TUBE-PD, right panel). Left panel: shows the input, representing 2.5% of the total protein extract. B) Western blot analysis of immE7 cells after expression of different HPV E6 proteins. ImmE7 cells were transfected with 2 µg of expression plasmids harboring the full-length sequence of E6 from HPV types 16, 18, 6 or 11. Cells were incubated for 24 h prior to protein extraction and Western blot analysis. C) qPCR analysis of IL-1β mRNA derived from genital wart biopsies positive for low-risk HPV6 (n = 5) using primary keratinocytes as a positive and SiHa cells as a negative control. Ordinate: expression as fold changes using SiHa cells as reference which was arbitrarily set as 1. D) For Immunoprecipitations (IP), SiHa cells were transfected with p53-YFP and pro-IL1β expression plasmids and treated with 10 µM MG132 for 6 h prior to protein extraction. Upper panel: p53 IP, middle panel: GFP-IP, lower panel: IL-1β IP. Western blot analyses were performed by incubating the membranes with antibodies against p53, E6-AP, IL-1β and HA. Input, representing 10 or 15% of the total protein extract.
(EPS)
